# Seroprevalence of Anti-SARS-CoV-2 Antibodies among Municipal Staff in the Municipality of Prishtina

**DOI:** 10.3390/ijerph182312545

**Published:** 2021-11-28

**Authors:** Bujar Gashi, Vesa Osmani, Rrezart Halili, Teuta Hoxha, Agron Kamberi, Nexhmedin Hoti, Riaz Agahi, Vlora Basha, Visar Berisha, Ilir Hoxha

**Affiliations:** 1 Main Family Medical Centre, Municipality of Prishtina, 10000 Prishtina, Kosovo; bujar.a.gashi@rks-gov.net (B.G.); rrezart.halili@omk-rks.org (R.H.); hoxhateuta@hotmail.com (T.H.); agronkamberi2@gmail.com (A.K.); hotinexhmedin5@gmail.com (N.H.); 2 Evidence Synthesis Group, 10000 Prishtina, Kosovo; vesa@evidencesynthesis.group; 3 Research Unit, Heimerer College, 10000 Prishtina, Kosovo; riaz.agahi@kolegji-heimerer.eu; 4 IndexKosova, 10000 Prishtina, Kosovo; V.Basha@indexkosova.com (V.B.); v.berisha@indexkosova.com (V.B.); 5 The Dartmouth Institute for Health Policy and Clinical Practice, Lebanon, NH 03766, USA

**Keywords:** COVID-19, Kosovo, seroprevalence, municipality of Prishtina, municipal workers

## Abstract

Background: Some studies have assessed the seroprevalence of anti-SARS-CoV-2 antibodies in different populations. Very few studies have explored seroprevalence in municipal workers, an important and potentially high-risk population. This study aims to determine the prevalence of anti-SARS-CoV-2 antibodies in municipal workers, with the additional examination of the association of prevalence with various demographic, health-related, and epidemiological factors. Methods: We surveyed and tested for seroprevalence 418 public servants from the municipality of Prishtina, the capital of Kosovo. The primary prespecified outcome was the seroprevalence of anti-SARS-CoV-2 antibodies, IgG, and IgM. Additional outcomes were crude and adjusted odds ratios of seroprevalence by different factors. Results: 21.1% of municipal workers tested positive for either IgM or IgG. Of these, 9.6% were positive for IgM and 19.4% for IgG. Data showed high levels of adherence to protective measures, e.g., social distancing in the office, but calculation of ORs did not show a significant difference between those reporting adherence to such measures and those reporting nonadherence. Of other examined factors, significantly lower odds were observed for smokers (0.52, 95% CI 0.28, 0.97), while municipal workers with infected family members had elevated odds of seropositivity according to both crude (2.19, 95% CI 1.34, 3.59) and adjusted (2.00, 95% CI 1.17, 3.41) ORs. Conclusions: Most answers from public servants demonstrated compliance to social-distancing policies in the workplace, but analysis of crude and adjusted odds ratios did not suggest a significant effect between municipal workers who followed these guidelines and those who did not. Results from this study help Kosovo policy makers in understanding the level of prevalence of COVID-19 in municipal workers and the effect of different factors on such prevalence. Results from the study could inform future decisions on the design and application of protective measures for municipal workers. Our findings should encourage further research to assess the extent of the spread of COVID-19 to other essential workers in Kosovo, including retail workers.

## 1. Introduction

Kosovo was among the last-hit countries in the region and Europe by the coronavirus pandemic caused by severe acute respiratory syndrome coronavirus 2 (SARS-CoV-2). Kosovo has a population of about 1.8 million, with the highest population density being in the capital city. The first cases were confirmed on 13 March 2020 [1]. Between January 2020 and 27 May 2021, the World Health Organisation reported that Kosovo had a total of 107,270 confirmed cases of coronavirus disease 2019 (COVID-19) with 2231 deaths [2].

The course of COVID-19 in Kosovo has not been studied much, but current information reveals huge disparities between clinical features and disease prognosis among laboratory-confirmed positive cases. To avoid contact, and thereby minimise the risk of infection and reduce costs for protective equipment, Kosovar medical institutions have taken various restrictive measures [3]. 

Public servants in the municipality of Prishtina play a vital role during the pandemic [4]. They are considered to be key in maintaining services and remedying pandemic-related issues [5]. Public servants are also a highly exposed population to the coronavirus pandemic, given the direct contact that these workers have with the public at large. With the rapid spread of the COVID-19 in Kosovo, especially in the city of Prishtina as a highly infectious or red zone, it is necessary to understand the risks of positive anti-SARS-CoV-2 antibodies, and the relationship among sociodemographic, health-related, and epidemiological factors [6,7,8]. Little-to-no research has been conducted on the relationship between the aforementioned factors in public servants, and particularly not in this specific population.

Seroprevalence is widely used as a measure of the number of individuals who have been infected, for example, using cross-sectional studies to approximate the spread of COVID-19 through the population. Studies were also used in estimating population infection levels of COVID-19 in various countries and areas [9,10,11,12,13]. A systematic review by Rostami and coworkers estimated global seroprevalence at 3.38% [14]. Studies also examined seroprevalence in vulnerable populations, including primary health workers [15,16,17], the homeless [7], and individuals with comorbidities [18]. Thus far, studies in Iran [13] and India [19] examined COVID-19 seroprevalence among study populations consisting of different professions, but we did not come across studies focusing on municipal workers. The level of exposure to SARS-CoV-2 by different professionals, and hence their COVID-19 seroprevalence, is likely to differ due to the type and amount of contact that they have with other people with whom they interact on a daily basis depending on their profession [20,21].

This study examines the prevalence of anti-SARS-CoV-2 antibodies among the municipal workers of the municipality of Prishtina, and assesses the influence of other variables, such as adherence to protective measures, on this relationship. We were primarily concerned with the estimation of COVID-19 prevalence, as that was the primary interest of the policy-making authorities. There were concerns about the extent to which municipal workers are exposed to the COVID-19 virus and infection. At the same time, we were interested to examine as many factors that may affect prevalence as possible, with special attention to protective measures. All this information could help policy makers in devising more effective measures for protection and the management of the situation.

## 2. Materials and Methods

### 2.1. Survey Details 

Two main sources of data were used. First, seroprevalence testing data. Two immunoassay tests were performed with the purpose of detecting IgM and IgG antibodies. The testing was carried out using VIDAS^®^ SARS-CoV-2 (IgM reference 423833) and VIDAS^®^ SARS-CoV-2 (IgG reference 423834) by bioMérieux. Testing was performed at the COVID-19 Testing Center of the Primary Care Network in Prishtina. Ongoing retrospective manual data abstraction was performed from the records of workers who had been tested for anti-SARS-CoV-2 antibodies between 1 October and 31 November 2020. The rest of the data were collected via cross-sectional survey that examined key information on health behaviour, epidemiological behaviour, family exposure, etc. Data collection was performed via online survey using the questionnaire (Supplementary Materials) and was carried out by a research firm in Prishtina.

### 2.2. Patient and Public Involvement

Research subjects were not involved in the research design, but participated with informed consent. This research was carried out with the informed consent of all participants, given freely before beginning the interview. This consent was given using the following procedure. Participants were informed that their participation was voluntary, and were given the option to refuse to answer any question or terminate the interview and participation at any time. All interviews were performed via telephone. Interviewers read and reviewed the informed consent describing the study purpose and all study procedures to the participants; after confirming the participants’ understanding of the informed consent, voluntary participation was elicited.

### 2.3. Sample Selection

**Figure 1:**
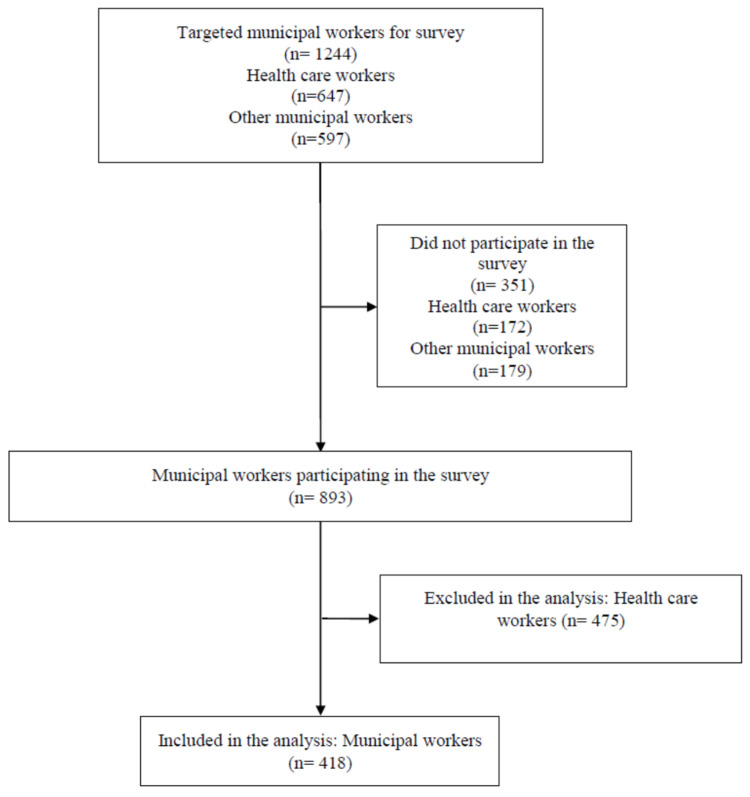
Sample selection process.

Participants of the study were identified via information provided by the municipality of Prishtina. The target sample of the municipal survey, was consecutive and intended to include all healthcare and administration workers of the municipality of Prishtina. The sample selected for this study was only composed of municipal workers (Figure 1). Out of 579 municipal workers, 179 did not participate because they refused, were on leave, were busy, missing data, or it was not possible to contact them due to no connection or a wrong phone number. The total sample size was 418 municipal workers in Prishtina.

### 2.4. Variables

The survey dataset contained key information on health behaviour (i.e., smoking) and status (i.e., existing conditions), and epidemiological behaviour (i.e., family exposure, adherence to protective measures, and similar variables). We identified, selected, coded, and recoded all variables related to COVID-19 symptoms in municipal workers who had tested positive for antibodies (IgM- or IgG-positive). We included other variables, such as blood group, which was examined as a pertinent variable in seroprevalence studies from other groups [22,23]. Apart from health determinants of these individuals, we also examined behavioural factors related to the context of Kosovo such as workspace behaviour and socialising during the relevant period. Data were collected by means of a standardised, precoded questionnaire (Supplementary Materials) that was locally pilot-tested prior to starting fieldwork. The survey questionnaire was designed to collect data on respondents’ sociodemographic characteristics, adherence to protocol and measures for protection against SARS-CoV-2, history of travel and contacts, evidence of symptoms, history of chronic diseases, recent history of consultations with medical professionals and potential treatment, household information on COVID-19 testing and symptoms, and treatment outcomes. The reason for collecting all this information was twofold: First, we ensured to collect information on all protective measures and behaviours that could determine seroprevalence, and could be relevant for adjusting existing and drawing new policy measures to protect municipal workers from infection. The other set of variables were included for control purposes, i.e., to make sure that we examined the prevalence and protective measures that shape such prevalence without confounding effects that could be due to other factors. we ensured to have means to control for the effect of variables that could influence the prevalence of COVID-19 on the basis of the best available evidence. This was conducted by consulting the literature and discussing with the expert group set to supervise this study. Vaccination for SARS-CoV-2 in the country only started at the end of March 2021 and hence was not considered in the data collection or analysis. 

Some of the variables that collected data on a scale (1–4 and 9 for do not know or refusal) were recoded to three categories. One category included “true” or “somewhat true”, and the other “somewhat untrue” or “not true”. The category of “do not know/refused” was left as it was as a separate category within those variables. 

### 2.5. Outcome Measures

The primary prespecified outcome was the seroprevalence of anti-SARS-CoV-2 antibodies IgG or IgM. A serological (IgM or IgG) test was considered positive when “i” (index value result with VIDAS^®^ SARS-CoV-2) was equal to or larger than 1. Prespecified secondary outcomes were crude and adjusted odds ratios of seroprevalence for a variety of demographic, health-related, and epidemiologic behaviours, and the circumstance variables described above.

### 2.6. Statistical Analysis

First, we examined seroprevalence for IgM and IgG antibodies. We then performed descriptive analysis of COVID-19 seroprevalence against several categories of variables, i.e., demographic, health-related, and epidemiological. Crude univariable logistic regression was performed to test the unadjusted associations of variables with the odds for seroprevalence. Then, all available variables representing demographic, epidemiological, and other behavioural characteristics that may play a role in COVID-19 prevalence and had a *p* value < 0.10 were included in the multiple logistic models. We performed a chi-squared test for each association. The study is reported according to the STROBE statement for observational studies [24]. Analyses were performed using Stata IC 15, StataCorp, TX, United States.

## 3. Results

### 3.1. Study Sample

Our study sample was composed of a total of 418 municipal workers working in Prishtina, the capital city of Kosovo. “Municipal workers” means employees of the municipality of Prishtina, i.e., public servants in a variety of areas. Administrative workers (29.4%) were the largest group, with some portion working in culture (12.7%), health (6.7%), and many other departments.

### 3.2. Seroprevalence of Anti-SARS-CoV-2 Antibodies

From our sample of 418 municipality workers, a total of 88 (21.05%) were positive for either IgG or IgM anti-SARS-CoV-2 antibodies. Of these, 40 participants tested positive for IgM antibodies (9.57%). A larger number (81 participants, 19.38%) tested positive for IgG antibodies (Table 1).

### 3.3. Effect of Different Factors on Seroprevalence Odds 

We analysed a range of relevant variables and their effect on seroprevalence by calculating the odds ratios (Table 2). Of primary interest was the effect of a variety of protective health behaviours relevant to the workplace. The vast majority of surveyed workers reported adherence to social-distancing protocols or hand hygiene (95.5% and 97.9%). Reported regular practice of hand hygiene was not significantly associated with an increase or decrease in seropositivity (crude—1.31, 95% CI 0.15, 11.38, adjusted—0.68, 95% CI 0.04, 11.54). Participants who had reported keeping of 2 m distance in the office were not associated with decreased odds of seropositivity, nor was a difference in odds associated with keeping of a 2 m distance in other areas of the workplace. The reported practice in the workplace of leave for workers who had tested positive for COVID-19, as self-reported by respondents, was also assessed but was not associated with a decrease in the odds of seropositivity. Presence in crowded workplace locations was another variable in the analysis that did not seem to affect the likelihood of seroprevalence. Lastly, the variable ‘careful office’, which was a measure of the overall level of adherence to protective measures, was not associated with a difference in odds ratio for seropositivity. 

We examined the effect of several control variables that could have affected the odds for seroprevalence. Municipal staff with infected family members had higher odds of seropositivity (crude—2.19, 95% CI 1.34, 3.59, adjusted—2.00, 95% CI 1.17, 3.41). Moreover, settlement size did not lead to a difference in the odds of seropositivity. Existing evidence showed correlation between the behaviour of family members and the prevalence of COVID-19 [25,26]. Our analysis measured the extent of the workers’ family members maximally respecting the protective measures. Our survey did not show a significant difference in odds for those who had reported family members respecting protective measures (crude—1.30, 95% CI 0.15, 11.28, adjusted—1.06, 95% CI 0.05, 21.55).

The other set of control variables were health status and behavioural variables. Among key health determinants within the sample population, analysis included the blood group of the municipal workers. There was variation in the odds ratios, for example, the crude and adjusted odds ratios for blood group B, in comparison with blood group O, were 1.72 (crude—95% CI 0.76, 3.94, adjusted—95% CI 0.70, 4.24). However, we did not observe a significant overall difference in the odds of seropositivity for different blood groups. Another key health determinant included in the analysis was the presence of existing health conditions among the municipal workers of the sample. The odds of seropositivity for those with pre-existing conditions were not significantly different (crude—0.50, 95% CI 0.23, 1.09, adjusted—0.59, 95% CI 0.26, 1.35). Varying global evidence since the beginning of COVID-19 has suggested a relationship between smoking and coronavirus prevalence or severity in different patients [27,28]. Analysis also included smoking as one of the health determinants among the municipal workers. Those who smoked had significantly decreased odds of seropositivity compared with nonsmokers (crude—0.42, 95% CI 0.24, 0.75, adjusted—0.52, 95% CI 0.28, 0.97). 

## 4. Discussion

The prevalence of COVID-19 among workers within the municipality of Prishtina included 88 of IgM- or IgG-positive cases (21.05%): 40 IgM-positive cases (9.57%), and 81 IgG-positive cases (19.38%). We also calculated crude and adjusted odds of seropositivity for other health-related and epidemiological variables, including blood group, existing conditions, smoking, and infected family members. According to our study, smokers had lower odds of seroprevalence than that those of nonsmokers, while municipal workers with infected family members had higher odds of testing positive for anti-SARS-CoV-2 antibodies. We did not observe significant differences in the odds of seropositivity for hand hygiene, family members respecting protective measures, distance in the office, leave from work after testing positive for COVID-19, presence in crowded workplace locations, and careful office environments.

### 4.1. Study Strengths and Limitations 

The content of the questionnaire was based on existing published studies and consultations with healthcare experts and the staff of the Main Family Medical Centre of Prishtina. Expert input from the private and public sectors into the study design, questionnaire, and overall progress of study is one of the primary strengths of this study. Our study also allowed for assessing the level of risk of this previously unexamined group of workers and the extent to which COVID-19 has spread. One limitation of the study is the small sample size, which was due to the relatively small population size. Overall, our understanding of the global levels of risk for municipal workers, and how this compares with seroprevalence in Kosovo, is hampered by a lack of similar studies for comparison. The calculation of odds ratios did not demonstrate any significant reduction in COVID-19 risk due to adherence to protective measures, distancing in offices, etc. The self-reported nature of the survey results may have led to measurement bias in the results. It is also uncertain how long anti-SARS-CoV-2 antibodies persist [29], and as such, municipal staff may have been previously infected but no longer have antibodies. Uncertainty about exactly how long antibodies remain in the bloodstream also means that comparisons of different studies may be convoluted by the study timeframe.

### 4.2. Context

Various studies examined the occupational health of frontline workers such as doctors, nurses, and the related healthcare personnel, highlighted the risk to health workers [30], and found varying prevalence levels, for example, 4.2% in an Egyptian study [31], or rates as high as 57% for female HCWs in the Democratic Republic of the Congo [32]. Similarly a report by Mutambudzi and coworkers reported an increased risk of severe COVID-19 for essential workers, with a risk ratio of 1.60 [20]. However, a limited number of studies observed topics specifically related to administrative, cleric, or municipal workers in the local public administration who are also exposed and of service to the public, and none of these studies estimated seroprevalence or risk among this group [33,34]. Fellows of the Collegium Ramazzini generally concluded that increased risk of COVID-19 infection is posed for workers with exposure and contact to infected persons and the public [35]. In this regard, the authorities should establish policies for high-risk workers that presume that all the COVID-19 infections of these workers are work-related, enforcing occupational health standards and drafting pandemic preparedness plans. Although not in the same sector, an analogy can be drawn with retail workers because of their direct interaction with the public in the course of their work. A study conducted with U.S. retail workers reported that employees who had direct contact with customers had an odds ratio of 5.1 for seropositivity for COVID-19 [26]. Another assessment of occupational risk conducted in Italy showed that occupations such as public administration and compulsory social security are in the medium-to-high risk group with respect to COVID-19 exposure [36]. 

Our results suggest that occupational adherence to distancing and hygiene protocols did not significantly affect the odds of seroprevalence. This contrasts other studies that reported a restrictive effect on viral transmission. For example, a systematic review by Chu and co-workers reported that compared with less than 1 m physical distancing, distancing of over 1, was associated with an odds ratio of 0.18 for transmission, while use of face masks was associated with an odds ratio of 0.15 for infection [37]. With this in mind, it is possible that an overall high level of adherence to protective measures, such as 2 m distance in offices, signifies an overall level of distance which affects all workers. A study by Fan et al., which examined different types of workers, including public servants in the category of white-collar workers, demonstrated a high level of knowledge about protective measures and a high level of adherence among these workers [38], which agrees with our results. In other words, the lack of difference between workers who followed protective measures and those who did not may be explained by the very high overall adherence among municipal workers giving a protective effect to all workers whether they observed protective measures or not.

The significant increase in the odds of seropositivity for those with infected family members suggests, however, that the risk of seropositivity primarily comes from outside the workplace, which again reflects the high levels of adherence to protective measures in municipal offices. We did not find a significant change in seroprevalence according to settlement size, although living in a densely populated area or in cramped housing conditions are risk factors for elevated seroprevalence [39]. Similarly, smoking was reported to be a significant risk factor for COVID-19 seroprevalence and risk of case severity, although it was not observed in our study [27,28]. A similar trend was observed for comorbidities, where conditions such as hypertension of diabetes were associated with increased seroprevalence [39,40], but we did not observe a significant difference in seroprevalence.

### 4.3. Policy Implications

Municipal workers may be at heightened risk of COVID-19 due to the nature of their work in public service, especially considering the difference between the proportion of seropositive municipal workers and the proportion of confirmed cases in the population of Kosovo [2]. A study by Møller highlighted the dilemma of public servants between the provision of services and self-protection [41], and this is clearly an issue that must be addressed by policy makers. Observations in several developing and developed countries highlighted the need for proper occupational health policies that specifically cater to this group of workers, and for maximal social distancing in lieu of vaccines—two paramount mechanisms against further COVID-19 infections among public servants. The results of this study indicate that protective measures enacted by municipal administrations have been vastly implemented. Although we could not learn much about the effect of different protective measures, policy makers in Kosovo and elsewhere should regularly follow emerging evidence and practices from other countries in order to continue to inform policies that shape the implementation of protective measures. One of most important directions considering vaccine availability is to ensure that all municipal workers are vaccinated. 

### 4.4. Research Implications

Future research could explore opportunities for a more objective measurement of various behavioural patterns of municipal workers. One option is the use of GIS devices to track the movements of municipal workers as a more objective way for tracking potential exposure to the virus, which was implemented in other research projects [42,43]. This could, for example, more objectively measure the 2 m distance among workers and clients, and several office-related exposure variables that we attempted to specify as self-reported measures in the survey questionnaire. This option was considered for a population prevalence study in Prishtina. Our findings should also spur further research to assess the extent of the spread of COVID-19 in municipal workers elsewhere, and to other essential workers in Kosovo, including retail workers.

## 5. Conclusions

The findings revealed quite a high prevalence among municipal workers at the end of first year of the pandemic in Kosovo. Whereas most of the answers from the public administration workers showed general compliance to social-distancing policies in the workplace, the calculation of odds ratios for such workplace behaviours did not reveal a difference in the odds of seropositivity. Results from this study help Kosovo policy makers in understanding the level of prevalence of COVID-19 in municipal workers and the effect of different factors on such prevalence. The results of this study could inform future decisions on the design and application of protective measures for municipal workers. Our findings should encourage further research to assess the impact and spread of COVID-19 to other essential workers in Kosovo, including retail workers. Policy makers should also follow other evidence that becomes available globally as results from new studies become available. One of most important directions considering vaccine availability is to ensure that all municipal workers are vaccinated.

## Figures and Tables

**Table 1:** Summary results of prevalence of COVID-19 among municipality workers.

	Events/Total; %
IgM- or IgG-positive	88/418; 21.05%
IgM-positive	40/418; 9.57%
IgG-positive	81/418; 19.38%

**Table 2:** Crude and adjusted odds ratios.

Variable ^a^	Positive	Negative	Crude Odds Ratio (95% CI)	*p* Value	Adjusted Odds Ratio (95% CI)	*p* Value
	Events/Total; %	Events/Total; %				
**Blood Group**						
O	26/88; 29.5%	106/330; 32.1%	Reference		Reference	
A	27/88; 30.7%	80/330; 24.2%	1.38 (0.75–2.54)	0.307	1.35 (0.70–2.61)	0.375
B	11/88; 12.5%	26/330; 7.9%	1.72 (0.76–3.94)	0.195	1.72 (0.70–4.24)	0.240
AB	3/88; 3.4%	18/330; 5.5%	0.68 (0.19–2.48)	0.559	0.57 (0.15–2.23)	0.420
Do not know/refuse	21/88; 23.9%	100/330; 30.3%	0.86 (0.45–1.62)	0.633	1.05 (0.53–2.06)	0.896
**With existing conditions**	8/88; 9.1%	55/330; 16.7%	0.50 (0.23–1.09)	0.082	0.59 (0.26–1.35)	0.211
**Smoking**	17/88; 19.3%	119/330; 36.1%	0.42 (0.24–0.75)	0.003	0.52 (0.28–0.97)	0.040
**Has infected family members**	37/88; 42.0%	82/330; 24.8%	2.19 (1.34–3.59)	0.002	2.00 (1.17–3.41)	0.011
**Settlement size**	178.6077 *	220.1931 **	1.00 (1.00–1.00)	0.023	1.00 (1.00–1.00)	0.028
**Hand Hygiene is Regularly Practiced**						
False	1/88; 1.1%	5/330; 1.5%	Reference		Reference	
True	85/88; 96.6%	324/330; 98.2%	1.31 (0.15–11.38)	0.806	0.68 (0.04–11.54)	0.791
Do not know/refuse	2/88; 2.3%	1/330; 0.3%	9.99 (0.40–250.42)	0.161	0.10 (0.00–19.27)	0.394
**Family Member that Respect Maximally Protective Measures**						
False	1/88; (1.1)	5/330; (1.5)	Reference		Reference	
True	84/88; 95.5%	323/330; 97.9%	1.30 (0.15–11.28)	0.812	1.06 (0.05–21.55)	0.969
Do not know/refuse	3/88; 3.4%	2/330; 0.6%	7.50 (0.46–122.70)	0.158	13.61 (0.16–1146.77)	0.248
**Keeping Distance of 2 m in the Office**						
False	9/88; 10.2%	48/330; 14.5%	Reference		Reference	
True	64/88; 72.7%	247/330; 74.8%	1.38 (0.64–2.96)	0.406	1.36 (0.51–3.65)	0.540
Do not know/refuse	15/88; 17.0%	35/330; 10.6%	2.29 (0.90–5.82)	0.083	0.59 (0.93–3.80)	0.581
**Keeping Distance of 2 m in Other Workplace Spaces**						
False	9/88; 10.2%	41/330; 12.4%	Reference		Reference	
True	63/88; 71.6%	262/330; 79.4%	1.09 (0.51–2.37)	0.817	1.00 (0.35–2.87)	0.998
Do not know/refuse	16/88; 18.2%	27/33;0 8.2%	2.70 (1.04–6.98)	0.041	3.23 (0.50–21.00)	0.220
**Leave from Work of Workers Positive with COVID-19 Practiced**						
False	3/88; 3.4%	7/330; 2.1%	Reference		Reference	
True	75/88; 85.2%	307/330; 93.0%	0.57 (0.14–2.26)	0.423	0.51 (0.10–2.52)	0.407
Do not know/refuse	10/88; 11.4%	16/330; 4.8%	1.46 (0.30–6.98)	0.637	0.65 (0.98–4.36)	0.661
**Presence in Crowded Workplace Locations**						
False	32/88; 36.4%	166/330; 50.3%	Reference		Reference	
True	27/88; 30.7%	98/330; 29.7%	1.43 (0.81–2.53)	0.219	1.33 (0.72–2.47)	0.362
Do not know/refuse	29/88; 33.0%	66/330; 20.0%	2.28 (1.28–4.06)	0.005	1.76 (0.90–3.41)	0.096
**Careful Office**						
False	3/88; 3.4%	21/330; 6.4%	Reference		Reference	
True	81/88; 92.0%	303/330; 91.8%	1.87 (0.54–6.43)	0.320	2.88 (0.59–14.02)	0.19
Do not know/refuse	4/88; 4.5%	6/330; 1.8%	4.67 (0.81–26.87)	0.085	3.45 (0.34–35.01)	0.294

^a^ For binary variables, reference if the value is false is, e.g., for smoking, 1 for true and 0 for false, which is reference value for the variable. * Mean. ** Standard deviation.

## Data Availability

Data are available on reasonable request.

## Supplementary Materials

The following are available online at https://www.mdpi.com/article/10.3390/ijerph182312545/s1. The questionnaire.
