# Supplementary material for: Seroprevalence of Anti-SARS-CoV-2 Antibodies among Municipal Staff in the Municipality of Prishtina
Source: Int J Environ Res Public Health. 2021 Nov 28;18(23):12545. doi: 10.3390/ijerph182312545 (PMC8656675; doi:10.3390/ijerph182312545)
Supplement: Supplementary file 1 [file ijerph-18-12545-s001.zip › ijerph-1451454-supplementary.pdf]

Supplementary Material
Questionnaire for the study about COVID-19
THIS PART OF INFORMATION IS COLLECTED FROM THE RESPONDENT BY PHONE

I. Sample entry and selection
** START SELECTING THE RESPONDENT BY VERIFIING HIS / HER IDENTITY FROM THE SAMPLE LIST **

1. INTRODUCTION. "HELLO! I AM ________________ AND I AM CALLING YOU ON BEHALF OF THE MUNICIPALITY OF PRISHTINA. ARE YOU [NAME AND SURNAME FROM THE LIST]? WE HAVE RECEIVED YOUR PHONE NUMBER FROM INFORMATION YOU GAVE WHEN YOU WERE TESTED FOR COVID-19 IN THE MUNICIPALITY OF PRISHTINA. THE MUNICIPALITY OF PRISHTINA IS CARRYING OUT A STUDY ABOUT THE SPREAD OF THE COVID-19 VIRUS AMONG THE WORKERS OF THE MUNICIPALITY OF PRISHTINA. ONE OF THE GOALS OF THE STUDY IS THE ANALYSIS OF THE RESULTS OF SEROLOGICAL TESTS. "

P1. CAN I GET YOUR CONSENT TO USE THE RESULT OF YOUR SEROLOGICAL TEST FOR STATISTICAL ANALYSIS? IN ACCORDANCE WITH INTERNATIONAL RESEARCH STANDARDS, YOUR TEST RESULT WILL NEVER BE RELATED TO YOUR PERSONAL INDETITY DATA. YOUR PERSONAL IDENTITY DATA WILL BE DISPOSED AFTER RESEARCH QUALITY CONTROL AND WILL NOT BE USED DURING STATISTICAL ANALYSIS.

1. *YES — CONTINUE*
2. *NO — END THE INTERVIEW AND CONTINUE WITH THE NEXT RESPONDENT*

"AS PART OF THIS STUDY I WOULD LIKE TO ASK YOU SOME QUESTIONS ABOUT YOUR HEALTH CONDITION AND YOUR EXPOSURE TO THE COVID-19 VIRUS. IF YOU HAVE A FEW MINUTES, PLEASE ANSWER THE FOLLOWING QUESTIONS. YOUR ANSWERS WILL BE COMPLETELY SECRET, IN ACCORDANCE WITH INTERNATIONAL RESEARCH STANDARDS. "

2. IF THE RESPONDENT REJECTS TO PARTICIPATE, CONTINUE TO THE NEXT RESPONDENT.

3. IF THE RESPONDENT CANNOT RESPOND AT THE MOMENT, TRY TO ASSIGN THE CALL FOR LATER IN THE DAY OR DURING THE NEXT WORKING DAYS. ENTER THE MOST SUITABLE DATE AND TIME FOR THE RESPONDENT.

II. Basic information about the respondent

M1. Respondent identification number from the sample list __ __ __

M2. In which municipality do you currently live?
1. Prishtina
2. Other municipality. WRITE:________________

M3. Is your place of residence urban or rural?
1. Urban
2. Rural

M4. IF YOU LIVE IN URBAN PRISTINA: In which neighborhood of Prishtina do you live?

1. Qendër
2. Bregu i Diellit
3. Ulpianë
4. Dardani
5. Lakrishte
6. Lagja e Muhaxherëve
7. Velani
8. Mati I
9. Lagja e Spitalit
10. Taukbashqe
11. Arbëri (Dragodan)
12. Vetërnik
13. Kodra e Trimave
14. Kolovicë
15. Kalabria
16. Tophane
17. Sofali
18. Tjetër ______________

III. Information about the respondent and his / her position

Q1. What is the highest level of education you have completed?	1. Primary<br>2. Secondary<br>3. University<br>4. Masters/PhD
Q2. Gender?	1. Female<br>2. Male
Q3. Year of birth __ __ __ __	
Q4. Do you know your blood type? If yes: What is your blood group?	1. A<br>2. B<br>3. AB<br>4. O<br>5. Don’t know – GO TO Q6
Q5. Do you know the RH factor of your blood type? If yes: What is the factor?	1. Rh+<br>2. Rh-<br>3. Don’t know
Q6. Which field do you work in?	1. Administration<br>2. Health<br>3. Social Welfare<br>4. Education<br>5. Culture<br>6. Sport<br>7. Agriculture<br>8. Finance<br>9. Property<br>10. Urbanism<br>11. Capital Investment and Contract Management<br>12. Public Services, Protection and Rescue<br>13. Cadaster<br>14. Inspection<br>15. Strategic Planning and Sustainable Development<br>16. Parks<br>17. MFMC/EMC
Q7. Are you a health worker?	1. Yes GO TO Q8<br>2. No SKIP TO Q9
Q8. What kind of health worker are you?	1. Doctor<br>2. Nurse<br>3. Technician<br>4. Administrative staff<br>5. Other, specify _________

IV. Information about the respondent's family and household economy

Q9. Household size:	|___|___| members
Q10. The size of the living space:	|___|___|___| square meters
Q11. Do you have a member of your household who is or has been infected with COVID-19, excluding yourself?	1. Yes GO TO Q12<br>2. No SKIP TO Q13
Q12. How many members of your household are or have been infected with COVID-19 excluding yourself?	__ __ members

Q13. Please evaluate whether each of these statements about you and your family members is true, somewhat true, somewhat untrue or not true:

	True	Somewhat true	Somewhat untrue	Not true	DK/Ref.
a. You and all family members wear masks properly (mouth and nose)	1	2	3	4	9
b. You and your family do not go to family gatherings and parties	1	2	3	4	9
c. You and your family do not visit the malls	1	2	3	4	9
d. You and your family do not visit gastronomy bars	1	2	3	4	9
e. You think your whole family has been careful and protected in every way from COVID-19	1	2	3	4	9

V. Information about health status

The following questions require answers regarding your health status.	
Q14. Do you currently suffer from any of these diseases? (read and circle)	1. Cancer<br>2. Chronic kidney disease<br>3. Chronic Obstructive Pulmonary Disease (COPD)<br>4. Weakened immune system from transplantation of solid organs<br>5. Obesity (overweight)<br>6. Serious heart conditions<br>7. Falciform cell disease (sickle cell)<br>8. Type 2 diabetes<br>9. None of the above
Q15. Do you currently suffer from other diseases? (read and circle)	1. Asthma (moderate to severe)<br>2. Cerebrovascular disease (affects the blood vessels and blood supply to the brain)<br>3. Cystic fibrosis<br>4. Hypertension or elevated blood pressure<br>5. Immune system weakened by blood or bone marrow transplantation, immune deficiencies, HIV, use of corticosteroids or use of other drugs that weaken the immune response<br>6. Neurological disorders, such as dementia<br>7. Liver disease<br>9. Pulmonary fibrosis (presence of lung tissue damage or injury)<br>11. Thalassemia (a type of blood disorder)<br>12. Type 1 diabetes<br>13. None of the above
Q16. FOR WOMEN: Are you currently pregnant?	1. Yes<br>2. No<br>3. Don't know
Q17. Do you smoke?	1. Yes<br>2. No

Q18. Please evaluate whether each of these statements is true, somewhat true, somewhat untrue or not true:					
	True	Somewhat true	Somewhat untrue	Not true	DK/Ref.
a. You have a high level of stress throughout the year	1	2	3	4	9
b. You try to live healthy by eating well, sleeping well	1	2	3	4	9
c. You have regular physical activity (like walking, physical work, recreation, sports, etc.) at least once a week	1	2	3	4	9
d. You go out regularly at least once a week	1	2	3	4	9

VI. Information about COVID-19 exposure in the office

Now, please explain the situation in the office.	
Q19. How many people are in the place / space / office where you work?	|__|__| persons
Q20. What is the size of the office?	|__|__| square meters<br>999. Don't know

Q21. Please evaluate whether each of these statements about you and your colleagues is true, somewhat true, somewhat untrue or not true:					
	True	Somewhat true	Somewhat untrue	Not true	DK/Ref.
a. Masks are held properly (mouth and nose) by your peers	1	2	3	4	9
b. Everyone maintains a 2-meter distance in the office	1	2	3	4	9
c. A 2-meter distance is maintained in other premises outside / besides the office	1	2	3	4	9
d. Work leave / stay at home is granted temporarily to persons who show symptoms	1	2	3	4	9
e. Work leave / stay at home is granted temporarily to persons who get tested and test positive	1	2	3	4	9
f. You and / or your colleagues stay together in crowded places, in places like buffet or any common part in the work environment	1	2	3	4	9
g. You think all your colleagues have been careful and you have been protected in every way by COVID-19	1	2	3	4	9

VII. Information regarding exposure to COVID-19 at work

ONLY FOR MUNICIPAL WORKERS.

Q22. Do you work with client/parties?	1. Yes<br>2. No	GO TO Q23<br>SKIP TO Q24
Q23. Are there protection panels when dealing with clients/parties?	1. Yes<br>2. No	
Q24. How many people do you meet during the day?	|__|__| persons	
Q25. How many days did you work from home in the period March-July 2020?	|__|__| day	

VIII. Information regarding exposure to COVID-19 at work

ONLY FOR HEALTH WORKERS.

Q26. Do you have regular contact with patients with COVID-19?	1. Yes<br>2. No<br>3. Don't know
Q27. Do you work in the testing or treatment unit of COVID-19 patients?	1. Yes<br>2. No
Q28. How many confirmed cases of COVID-19 have you had contact with?	|__|__| persons 999. Don't know
Q29. How many suspected cases of COVID-19 have you had contact with?	|__|__| persons 999. Don't know
Q30. Have you previously been diagnosed with a nasal sample test (PCR)?	1. Yes<br>2. No
Q31. Have you had COVID-19 compatible symptoms within the last 6 months?	1. Yes<br>2. No
Q32. Did you get the flu vaccine in 2019?	1. Yes<br>2. No

IX. Information about your behavior towards safeguards

Q33. Please evaluate whether each of these statements is true, somewhat true, somewhat untrue or not true for you personally:

	True	Somewhat true	Somewhat untrue	Not true	DK/Ref.
a. You hold the mask properly (mouth and nose)	1	2	3	4	9
b. You keep a 2-meter distance from everyone in the office	1	2	3	4	9
c. You keep a 2-meter distance in other premises outside / besides the office	1	2	3	4	9
d. You wash your hands regularly (several times a day) with soap or disinfectant	1	2	3	4	9
e. You have been provided with information materials or similar information about COVID-19 by officials or management	1	2	3	4	9
f. You have read those informative materials in detail	1	2	3	4	9
g. You think you have been careful and protected in every way by COVID-19	1	2	3	4	9

X. Information about the infection

Q34. If you have had COVID-19, what symptoms have you had? MULTIPLE ANSWERS	1. Temperature<br>2. Headache<br>3. Sore throat<br>4. Coughing<br>5. Muscle pain (myalgia)<br>6. Diarrhea<br>7. Fever<br>8. Difficulty breathing<br>9. Fatigue<br>10. New loss of taste or smell<br>11. Congested and leaking noses<br>12. Pain / vomiting<br>13. Didn't have symptoms<br>14. Didn't have COVID-19<br>15. Other: ____________________

M-5. Interviewer's code __ __ __ __
